# Aging diastole - root cause for atrial fibrillation and heart failure with preserved ejection fraction

**DOI:** 10.20517/jca.2024.22

**Published:** 2024-12-23

**Authors:** Markus Meyer, Julio Núñez, Parag Goyal, Daniel N. Silverman, Jop H. van Berlo, Valmiki Maharaj

**Affiliations:** 1 Lillehei Heart Institute, Department of Medicine, University of Minnesota, Minneapolis, MN 55455, USA.; 2 Cardiology Department, Hospital Clínico Universitario, INCLIVA, Universitat de València, València 46010, Spain.; 3 Program for the Care and Study of the Aging Heart, Department of Medicine, Weill Cornell Medicine, New York, NY 10021, USA.; 4 Division of Cardiology, Department of Medicine, Medical University of South Carolina, Charleston, SC 29425, USA.

**Keywords:** Heart failure, heart rate, ejection fraction, diastolic function, cardiac filling pressures, cardiac remodeling

## Abstract

The age-related decline in diastolic function can result in heart failure with a preserved ejection fraction (HFpEF) and atrial fibrillation (AF), which are comorbid conditions that are increasingly prevalent and have a high socioeconomic burden. In humans, diastolic dysfunction results from structural and functional changes that increasingly impede diastolic filling after midlife. Comorbidities and pathomechanisms that lead to additional increases in cardiac filling pressures accelerate the age-related deterioration in diastolic function. It is, therefore, that targeting the accelerators of diastolic dysfunction holds the most promise in reducing the risk for HFpEF and AF.

## Graphical Abstract

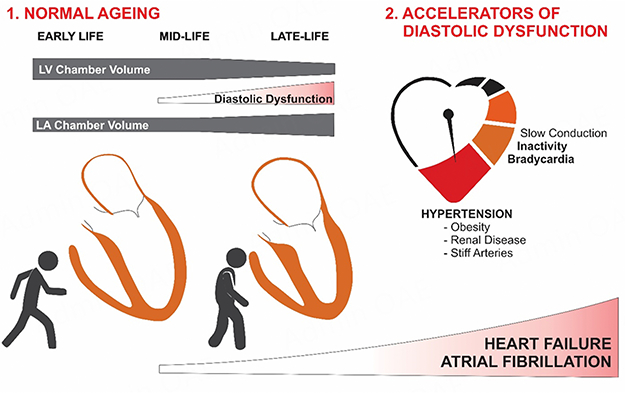

## INTRODUCTION

Diastolic dysfunction predisposes to heart failure with preserved ejection fraction (HFpEF) and atrial fibrillation (AF), which are prevalent, interdependent, and recurring comorbidities that reduce quality of life and survival. Both conditions share common risk factors, chief among them older age and the presence of hypertension, which adversely affect cardiac filling dynamics. We will first discuss the age-related changes in human cardiac structure and diastolic function and how they are modified. As the data contained in the literature often cover isolated findings or mechanisms, we rather focus on established patterns in aging that we deem most relevant to the deterioration in diastolic function. A better understanding of the intersecting pathophysiology of HFpEF and AF reveals therapeutic targets that can address both conditions.

## BASIC CONSIDERATIONS

Diastole refers to the phase of the cardiac cycle when the heart relaxes and the chambers are refilled with blood, which is a key prerequisite to normal cardiac function. The atria are exposed to ventricular pressures for most of the cardiac cycle, which helps explain why AF and HFpEF are closely linked. Ventricular relaxation is not passive recoil but is governed by energy-consuming processes within cardiomyocytes that remove calcium ions from the myofilaments. This actively lengthens muscle fibers to create a lower-pressure environment that promotes ventricular filling by suction. Cellular regulatory mechanisms, such as the phosphorylation of proteins involved in muscle contraction, modify diastolic function greatly. In late diastole, atrial contraction boosts ventricular filling and results in a modest amount of passive ventricular distension. However, it is ultimately the arterial blood pressure that provides the necessary hydrostatic force to maintain positive venous and atrial pressures that allow ventricular filling.

The pressure in the left atrium is a surrogate for cardiac filling pressure that closely correlates with the cardinal symptom of impaired diastolic function, that is, shortness of breath. Changes in cardiac structure and the myocardial substrate, such as concentric hypertrophy and fibrosis, can adversely affect diastolic function. These relationships are further influenced by the varying circulatory demands, which change venous and arterial loads and are modulated by heart rate, the principal regulator of cardiac output. Cardiac output reserve is directly proportional to the ability to exercise, which is among the best predictors of life expectancy^[1,2]^. Before discussing the natural deterioration in diastolic function over a lifespan, we will review how diastolic function is assessed in clinical practice.

### Assessment of diastolic function and HFpEF

The diagnosis of diastolic dysfunction and HFpEF is multifaceted and not straightforward. In 2022, the last AHA/ACC/HFSA practice guideline for the management of heart failure was released, replacing the 2013 guidelines^[3,4]^. In addition, in 2023, an expert consensus decision pathway for HFpEF was published^[5]^. These documents aimed to simplify the diagnosis and management of diastolic dysfunction and HFpEF.

What makes the diagnosis of HFpEF so difficult is that, despite its high prevalence, there is no perfect test to assess diastolic function. In patients with clinical symptoms consistent with volume overload or unexplained dyspnea, after a thorough history and physical exam, a transthoracic echocardiogram is the next step. If the ejection fraction is 50% or higher, HFpEF may be suspected. Important to the diagnosis of HFpEF is some evidence of increased filling pressures. Suggestive findings on echocardiography are the presence of enlarged atria, concentric ventricular remodeling, elevated pulmonary pressures, and a reduction in the tissue Doppler indices of relaxation^[6]^. However, evidence of diastolic dysfunction by echocardiography alone is insufficient to ascertain a diagnosis of HFpEF, and patients may still have HFpEF without abnormal diastolic function by echocardiography.

The gold standard for filling pressure elevations is a heart catheterization, which can be performed using one of two methods: right heart catheterization with pulmonary capillary wedge pressure measurement, or left heart catheterization with an assessment of left ventricular end-diastolic pressure. Increased filling pressures greater than 15 mmHg at rest, or if necessary with exercise (> 20 mmHg) or a fluid challenge (> 18 mmHg), are considered diagnostic if signs and symptoms of HFpEF are present. In routine clinical practice, invasive testing is rarely performed^[7]^. Thus, other diagnostic means have been evaluated to help predict if higher filling pressure and HFpEF are present. Besides echocardiography, cardiac biomarkers and clinical risk scores have predictive value.

The most relevant cardiac biomarkers used for the clinical assessment of diastolic function and HFpEF are natriuretic peptides: B-type natriuretic peptide (BNP) and N-terminal pro-BNP (NT-proBNP). BNP and NT-proBNP (a by-product of the cleaved prohormone proBNP) are primarily released by the ventricles in response to increased wall tension and strain, first and foremost by pressure and/or volume overload^[8]^. The natriuretic peptides are hormones that modify salt and water homeostasis, as well as blood pressure, and have diuretic, natriuretic, and vasodilator properties. Heart failure is associated with increases in natriuretic peptide blood levels, and measuring them by a blood draw can help ascertain or exclude HFpEF. The degree of natriuretic peptide elevation is linked to the severity of heart failure and has prognostic implications^[9]^. Here, it is noteworthy that patients with HFpEF tend to have lower levels of natriuretic peptides than patients with heart failure and a reduced ejection fraction (HFrEF), especially if they are obese^[8,10]^. In other words, normal natriuretic peptide levels in an obese patient do not preclude diastolic dysfunction or HFpEF. On the contrary, levels may be elevated in senescence and renal dysfunction without clinical evidence of HFpEF.

Given that HFpEF is a clinical diagnosis, scoring systems have been developed to provide physicians with a diagnostic probability based on patient history, demographics, and objective testing. The two scoring systems are the H_2_FPEF and the HFA-PEFF algorithms^[6,11]^. The H_2_FPEF score includes six components scored between 1–3 points, and these include: heaviness (BMI > 30 kg/m^2^), hypertension (2 or more blood pressure medications), presence of AF or pulmonary hypertension (pulmonary artery systolic pressure > 35 mmHg on screening echocardiography), elderly (age > 60 years), and an E/e′ ratio above 9 on Doppler echocardiography. A composite score of 6 or greater indicates a more than 90% likelihood that a patient has HFpEF.

The HFA-PEFF algorithm, developed by the Heart Failure Association of the European Society of Cardiology, is more involved but includes guidance regarding patients with intermediate probabilities and additional advice on how to screen for diseases that mimic HFpEF^[6]^. The first step involves a patient’s pretest probability based on clinical signs and symptoms of heart failure, patient demographics, risk factors, and comorbidities, as well as findings on screening echocardiography. The second step involves extracting specific echocardiographic parameters and using natriuretic peptides to assign a score to each patient. These echocardiographic parameters include functional tests such as tissue Doppler velocities and the presence of pulmonary hypertension, as well as structural measures such as left atrial volume, left ventricular mass, and wall thickness. Natriuretic peptide levels are stratified based on the presence or absence of atrial fibrillation. Scores of 5 or greater are considered diagnostic for HFpEF. Scores of 2–4 have an intermediate probability for HFpEF and additional testing is recommended as a third step. These tests include a diastolic exercise stress echocardiogram or heart catheterization. Finally, step 4 is meant to exclude conditions that mimic HFpEF, such as infiltrative or restrictive cardiomyopathies. Additional testing may include advanced imaging, i.e., cardiac magnetic resonance imaging, cardiac computed tomography, positron emission tomography, or nuclear scintigraphy. Depending on the findings, endomyocardial biopsies may be warranted, and genetic testing may be advised. Once mimicking conditions are ruled out, the diagnosis of HFpEF can be made.

## CONTRIBUTORS TO AGING-RELATED DIASTOLIC DYSFUNCTION

### Changes in cardiac structure after midlife

**Figure 1.**
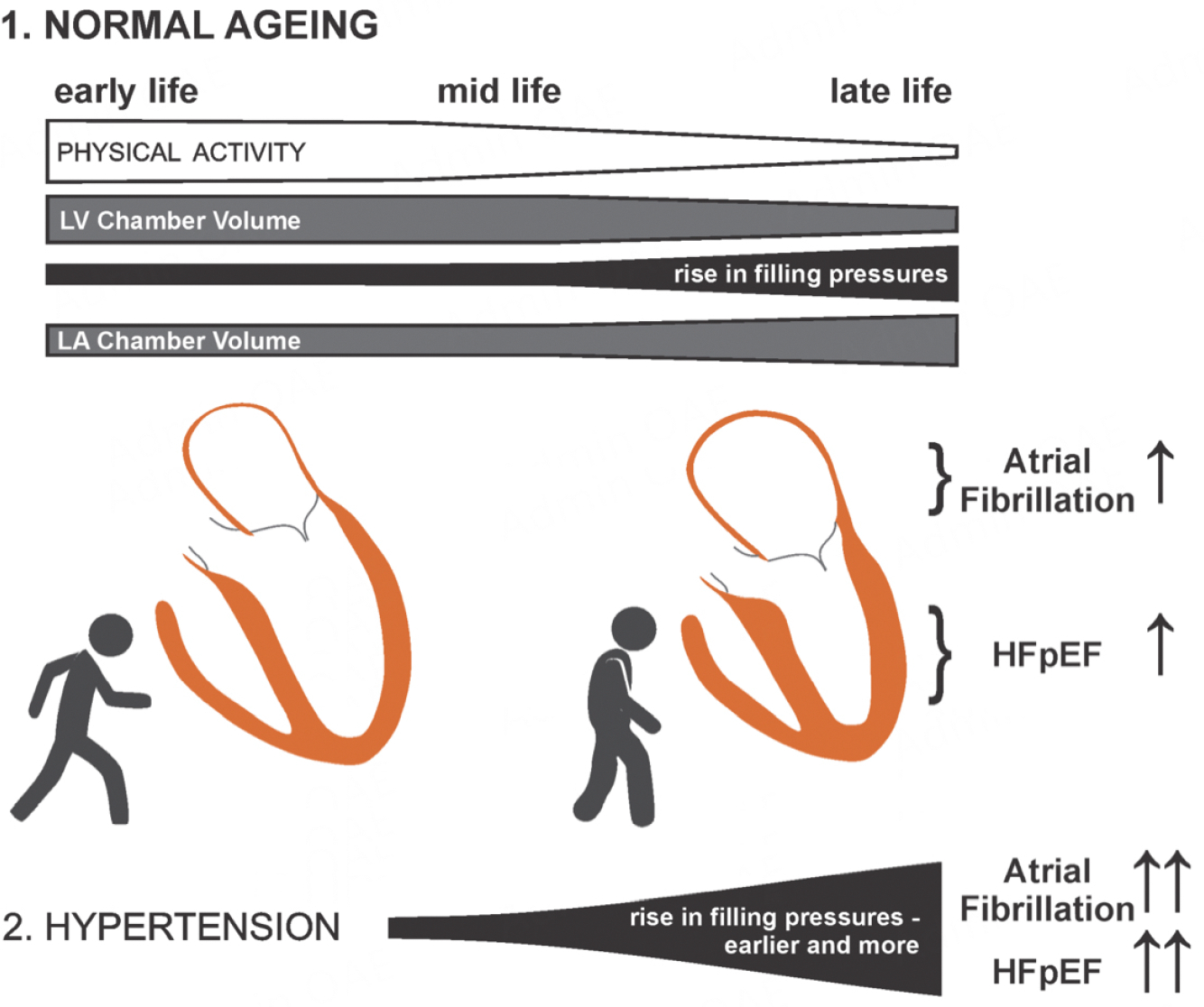
Age-related changes in cardiac structure and filling pressures. Hypertension has an accelerating effect on the rise of filling pressures with aging.

It is commonly overlooked that normal aging is associated with an unfavorable structural remodeling of both atria and ventricles [Figure 1]^[12–15]^. In midlife, left ventricular volumes start to regress by about 1% per year^[13]^. As ventricular mass does not change much with age, wall thickness increases, most notably in the basal septum, which reduces ventricular distensibility^[12]^. When combined with a lower chamber capacitance, this reduces functional reserve capacity and exercise tolerance^[16]^. In older adults, these changes are often detected by echocardiography, sometimes with evidence of diastolic dysfunction^[17]^. While ventricular volumes regress and diastolic function decreases with aging, atrial volumes increase primarily due to restricted ventricular filling^[15]^. These insidious and progressive changes not only reduce exercise capacity with aging but are also accompanied by measurable elevations in filling pressures that can manifest as dyspnea on exertion^[18–21]^. The increasing cardiac congestion contributes to venous remodeling with an associated expansion of the dependent venous blood pool that, when recruited to the central circulation during physical activity, overwhelms the heart rate-mediated contractile response with a build-up of diastolic myocardial tone that is pathognomonic for advanced diastolic dysfunction^[22–28]^. The resulting age-associated rise in cardiac filling pressure is the principal risk factor for AF and HFpEF. This process is hastened by arterial hypertension and reflected peripheral pressure waves that add to unfavorable structural remodeling^[29]^. This is evident in HFpEF patients, where smaller ventricular volumes are predictive of low exercise tolerance^[30]^.

### Exercise deficiency

Physical inactivity is an important contributor to age-related adverse cardiac remodeling, as it has been demonstrated that exercise is protective and can partially reverse ventricular volume loss and stiffness^[16,31]^. Additional evidence supporting the notion of exercise deficiency as an important contributor comes from echocardiographic studies of middle-aged patients after spinal cord injury. Compared to age-matched physically active individuals, calculated ventricular volumes are reduced by almost 20% among those with spinal cord injury, and the basal septum is thicker, which leads to reduced ventricular capacitance and compliance, notably in the absence of hypertension^[32]^. This increases the ventricular mass-to-volume ratio, which is a strong predictor of heart failure^[13]^. Although atrial dimensions were not reported in this study, early diastolic myocardial relaxation velocities were significantly reduced, suggestive of diastolic dysfunction, and explaining why paraplegic patients have a more than 3-fold increased risk for developing AF and heart failure^[33,34]^. A loss of left ventricular volumes has also been reported after prolonged space flight and extended bed rest, further supporting this notion^[35]^.

In contrast, hypertensive heart disease manifests as ventricular hypertrophy with an increase in mass. The increased wall thickness can lead to additional reductions in ventricular volumes. When superimposed on age-related structural remodeling with a reduction in diastolic function, this can result in persistent and more pronounced elevations in filling pressures at rest and on exertion, reflective of heart failure. At this stage, some patients may start to feel the urge to elevate their chest position when recumbent, which reduces filling pressures, thereby alleviating shortness of breath. This explains why patients with advanced HFpEF frequently use several pillows at night or prefer sleeping in a reclining chair.

### Electrophysiologic factors

The slowing in electrical conduction and subclinical sinus node dysfunction with aging also contribute to the decline in diastolic function and the progression of symptoms^[36,37]^. It is well established that the maximum predicted heart rate with exercise decreases markedly with age and the electrocardiographic P wave duration, PR interval, and ventricular conduction and repolarizations tend to widen^[36]^. While reduced peak heart rates play a direct role in decreasing exercise capacity, slowed electrical conduction will also contribute to diastolic dysfunction and higher filling pressures by adversely affecting mechanical synchrony and atrioventricular coupling^[36,38]^. Sinus node dysfunction with bradycardia will have an even more substantial role as it markedly raises filling pressures^[39]^. Heart rate variability also decreases with aging in part due to a withdrawal of vagal tone - this would be expected to result in higher resting heart rates after midlife, but this is not the case^[36]^. Longitudinal studies and larger cross-sectional population studies suggest a continued decrease in heart rates from adulthood into senescence^[40–42]^. Low heart rate-mediated increases in filling pressures may play a previously unrecognized role in the progression of diastolic dysfunction that will be discussed later.

## ACCELERATORS OF THE AGING-DEPENDENT IMPAIRMENT IN DIASTOLIC FUNCTION

### Hypertension

**Figure 2.**
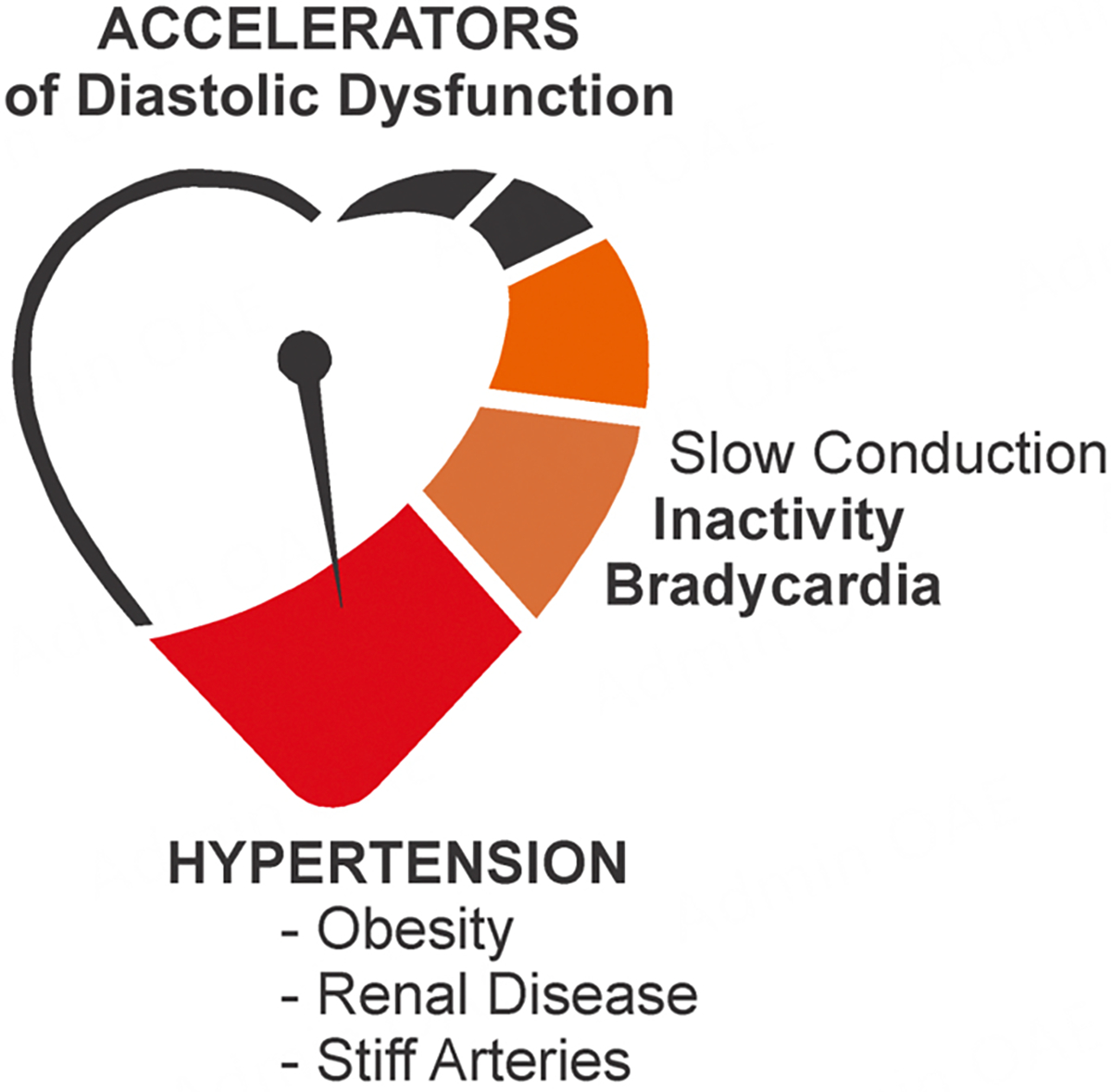
Accelerators of the age-related decline in diastolic function.

As discussed, hypertensive heart disease is commonly associated with HFpEF and AF as it accelerates the progression of diastolic dysfunction from the codependent deterioration of the ventricular and atrial substrates superimposed on physiological aging [Figure 2]^[43]^. This often manifests as an inappropriate rise in filling pressures on exertion or a volume challenge and is followed by sustained filling pressure elevations that permanently increase the atrial load^[44]^.

Hypertension also directly contributes to increased filling pressures, and explains why HFpEF and AF in midlife are becoming more common and blood pressure reductions are beneficial^[45,46]^. The SPRINT hypertension trial, which compared a systolic blood pressure goal of 120 to 140 mmHg, was stopped prematurely for the benefits of the more intensive blood pressure reduction^[47]^. This benefit was predominantly driven by the decrease in incident heart failure [HR (95%CI) 0.62 (0.45–0.84)] with a separation of the cumulative primary event curves after one year. In other words, an optimal blood pressure of 120 mmHg prevents heart failure compared to a moderately elevated blood pressure of 140 mmHg.

Unsurprisingly, lower blood pressures also resulted in less AF in the SPRINT trial [HR (95%CI) 0.74 (0.56–0.98)]^[48]^. In our clinical experience, it appears that the duration of hypertension plays an additional role in the development of AF and HFpEF. This would explain why relatively modest but long-term elevations in blood pressure, i.e., below 140 mmHg, appear sufficient to accelerate the deterioration in diastolic function.

### Obesity-driven hypertension

The impact of obesity on blood pressure is strikingly obvious in the weight-loss trials of glucagon-like peptide 1 (GLP-1) agonists. For example, tirzepatide resulted in a 15% reduction in mean weight over 1.4 years that lowered systolic blood pressure by 6.2 mmHg, easily surpassing the combined blood pressure effects of sacubitril-valsartan and sodium-glucose cotransporter-2 (SGLT-2) inhibitors in HFpEF trials^[49–52]^. In a phase-2 study of the GLP-1 agonist retatrutide, weight loss was nearly 25% with systolic blood pressure reductions of 12 mmHg in the high-dose group despite a concomitant withdrawal of antihypertensive medications^[53]^. It is, therefore, predictable that GLP-1 agonists will be highly effective in reducing incident HFpEF and AF by reducing filling pressures. A first glimpse of the tremendous potential of GLP-1 agonists was provided in the STEP-HFpEF trial that demonstrated a marked improvement in quality of life and reductions in clinical events^[54]^. The between-group difference primarily reflected the lower number of cardiac events in the semaglutide group (7 [2.7%] *vs*. 30 [11.3%] in the placebo group; *P* < 0.001), with 13 occurrences of heart failure and 12 of AF with placebo *vs*. 0 and 3 events in the semaglutide arm.

In clinical studies of HFpEF, about half of the patients have AF, and many with incident AF later develop HFpEF^[55–58]^. A majority of these patients have hypertension and receive blood pressure medications, and it is important to acknowledge that, despite the common use of antihypertensive agents, blood pressures in HFpEF outcome trials continue to be elevated^[50,52,58]^.

More evidence for the propinquity of AF and obesity in contemporary HFpEF cohorts is embedded in the H_2_FPEF diagnostic score. The presence of AF weighs heaviest in predicting HFpEF, followed by a body mass index above 30 kg/m^2[11]^. It is unsurprising that the prevalence of hypertension (45%) and obesity (42%) are similar in the US. At present, overindulgence is the main accelerator of diastolic dysfunction and explains the rise in AF and HFpEF^[58]^. This is commonly described as the cardiometabolic HFpEF and AF phenotype.

### Renal impairment-driven hypertension

In chronic renal disease, kidneys gradually lose their ability to effectively filter waste products and fluids. This results in fluid retention, causing an increase in blood volume and blood pressure that increases the risk for HFpEF and AF.

Chronic blood pressure elevations from any cause will eventually become detectable by cardiac imaging as hypertensive heart disease^[43]^. As discussed, the functional and structural ventricular and atrial abnormalities that reflect diastolic dysfunction include impaired atrial emptying, concentric left ventricular remodeling, or overt hypertrophy and left atrial dilation, to name a few^[43,55]^. Medication classes that are highly effective in HFrEF did not provide the same benefits in HFpEF, resulting in two decades of neutral clinical trials. This led to speculation that other pathomechanisms or conditions may have confounding roles in HFpEF^[59]^. The two most prominent non-blood pressure-driven conditions that may present as HFpEF will be discussed next.

### Hypertrophic cardiomyopathy and cardiac amyloidosis

Hypertrophic cardiomyopathy (HCM) and cardiac amyloidosis (CA) also accelerate the age-dependent deterioration in diastolic function. Suspicion for either condition should be highest among younger HFpEF patients without a history of hypertension. It is also important to recognize that more than half of individuals after the age of 70 years will have microscopically detectable “senile” amyloid deposits of unknown clinical significance that, in all likelihood, will contribute to the age-dependent deterioration in diastolic function^[29]^.

As stated earlier, when signs and symptoms of heart failure are present, due diligence is necessary to exclude both non-cardiac and cardiac mimickers of HFpEF. Of the cardiac causes, the most common mimickers include infiltrative or restrictive cardiomyopathies, pericardial diseases, or valvular heart disease. Echocardiography is a recommended starting point, whereas a “diagnostic shotgun” approach that employs every possible test is not helpful. A targeted approach is recommended that considers the clinical presentation within a patient’s demographic and age range, and assessment of associated non-cardiac manifestations. After which, potential testing, which may include specific laboratory tests, advanced imaging, heart catheterization, and endomyocardial biopsy, can be considered^[60]^.

HCM should be considered when there is unexplained left ventricular hypertrophy, especially with a family history of congestive heart failure or sudden cardiac arrest. Frequently, the diagnosis is suspected when left ventricular outflow tract obstruction or asymmetric hypertrophy is demonstrated by echocardiography. Additional cardiac magnetic resonance imaging can provide superior myocardial resolution with quantification of fibrosis, which adversely affects prognosis. Genetic testing can support the diagnosis in up to half the patients but also may have prognostic implications for affected individuals and their family members. Typically, endomyocardial biopsies have no role in the diagnosis of HCM.

CA is due to misfolded proteins that then form fibrils that infiltrate the myocardium. 95% of CA is either from misfolded mutant or wild-type transthyretin (TTR) protein, or light chain (AL) fragments from immunoglobulins and plasma cell dyscrasia^[61]^. While the incidence of AL CA is rather low (about 10 cases per million person-years), TTR CA can be rather common, possibly affecting about 13% of 60-year-olds with symptomatic HFpEF^[62]^. As amyloid is a systemic syndrome due to protein infiltration, extracardiac involvement is common and patients often present with a constellation of symptoms. Frequent signs and symptoms involve the musculoskeletal system, such as bicep tendon rupture, bilateral carpal tunnel syndrome, and lumbar spinal stenosis, to name a few. Patients with unexplained left ventricular hypertrophy, abnormal tissue Doppler on echocardiography, and low voltage electrocardiograms should be considered for CA screening, especially if extracardiac signs are present. Diagnosis of CA involves screening for AL disease with serum and urine protein immunofixation of antibody fragments, and performing bone scintigraphy, which can reveal an inappropriate myocardial uptake that is diagnostic for TTR disease. Treatment involves addressing the underlying cause, requiring a multidrug chemotherapy regimen for AL disease that may include autologous bone marrow transplant, or using protein stabilizers and silencers for wild-type or hereditary TTR disease.

In the next paragraph, we will critically reflect on mainstream research trends and misperceptions that, in our opinion, do not advance the understanding of diastolic dysfunction and are unlikely to result in treatments.

### Etiologic distractors

HFpEF is a heterogeneous syndrome and it has been argued that non-cardiac factors and comorbidities are responsible for the many neutral trials^[59,63]^. We posit that the pathophysiology of HFrEF is even more heterogeneous and that the efficacy of sodium-glucose cotransporter-2 (SGLT-2) inhibitors across most HFpEF subpopulations substantially weakens the heterogeneity argument. It is true, however, that HFpEF, in large part due to age and hypertension as precipitating factors, has many comorbidities. Nevertheless, European and American funding agencies decided to prioritize research efforts into the phenotyping of HFpEF, which, in our opinion, will lead to a wealth of statistical associations but no actionable items that will actually benefit patients^[64]^.

Efforts in the basic sciences to advance the understanding of HFpEF tend to focus on single, often arbitrary, mechanisms with the most innovative techniques, which for decades have not yielded a treatment for heart failure. At present, inflammation is commonly discussed as a cause for HFpEF, a hypothesized disorder of the myocardium and/or vasculature^[65]^. We are not aware of a clear link between inflammation and HFpEF. Additionally, in ventricular myocardium obtained from patients with HFpEF, there is no evidence that proinflammatory pathways and genes are activated, neither in the subendocardial myocardium nor in the epicardial myocardium^[66,67]^. The fact that obesity is generally accompanied by elevations in C-reactive protein should not be taken as evidence that inflammation causes diastolic dysfunction and HFpEF.

The track record of the publicly funded translational research enterprise is disappointing, to say the least, considering the large expense to taxpayers. Overbearing research bureaucracies, consensus-seeking review panels, and unproven selection mechanisms seem unable to turn discovery into health. To our knowledge, none of the proposed cures for rodent heart failure that utilized cutting-edge methods and were published in the most prestigious science journals were ever translated into a treatment. We surmise that artificial intelligence, the savior de-jour, may also not do the trick and we contend that normal human intelligence can prevail. A holistic view of the most promising treatment targets with multimodal therapeutic approaches that can address related defects will succeed or emerge by chance, as is the case with SGLT2 inhibitors.

In the following, we will discuss pathomechanistic themes and patterns that, based on available human data, play likely roles in the age-related deterioration of diastolic function to suggest realistic treatment targets.

## DETERMINANTS OF DIASTOLIC DYSFUNCTION

### Myocardial stiffness and fibrosis

As discussed, structural changes during normal aging alter the basic cardiac physical properties, which in itself will contribute to a rise in filling pressures. The changes in cardiac structure are paralleled by an age-related increase in myocardial fibrosis, predominantly by the increased deposition of collagen 1 and 3, that reduces tissue compliance to add to the progressive diminution in cardiac functional reserve^[68,69]^. Although the echocardiographic literature suggests an increase in ventricular mass with age, more accurate measurements by magnetic resonance imaging revealed the opposite, a small reduction in ventricular mass^[13]^. This discordance is explained by the age-related focal increase in basal septal thickness, which is a key measurement in mass estimation by echocardiography. Nevertheless, fibrosis increases with age, most likely in response to higher central blood pressures and, to a lesser extent, to replace lost cardiac myocytes. It is plausible that age-related arterial stiffening plays a contributing role by stimulating myocardial stretch mechanisms that initiate myokine signaling or activation of the angiotensin II/TGF-beta and endothelin-1 pathways that result in excess extracellular matrix production^[68,70–72]^. Other mechanisms that have been put forward are the effects of a more pro-inflammatory milieu in older hearts with radical oxygen species and the age-related decline in the turnover of collagen fibrils accompanied by increased cross-linking^[40,73]^. Such modified collagen may be less accessible to degradation mechanisms that balance extracellular matrix production and breakdown in response to demand-driven physiologic cardiac adaptations. It is clear from animal models and in patients that if hypertension is added to aging, fibrosis burden increases above what is normal for the respective age group^[40]^. In addition, hypertension will lead to a thickening of cardiac myocytes that at the organ level manifests as concentric hypertrophy with an increase in mass and stiffness. Left ventricular tissue samples of patients with hypertensive heart disease and overt HFpEF reveal a more than 3-fold increase in passive stiffness in stretched myocardium compared to age-matched control myocardium, with variable increases in total collagen expression and content^[67,69,74]^.

### Adrenergic signaling and protein hypophosphorylation

Another theme in aging and HFpEF is the attenuated adrenergic response leading to cellular hypophosphorylation^[75]^. Protein phosphorylation is a crucial biochemical process whereby kinases and phosphatases add or remove phosphate groups to cellular proteins. This process serves important functions such as regulation of enzyme activity, signal transduction, cellular communication, metabolic regulation, and gene expression. In the heart, basic functions such as contractility, sinus node activity, and conduction velocity are regulated by phosphorylation. Basal myocardial stiffness is also affected. A first clue that hypophosphorylation may play a role in HFpEF was provided in isolated myocytes of patients^[76]^. Administration of protein kinase A lowered passive stiffness more than in control cells. This effect was linked to titin, a giant cytoskeletal protein that changes its stiffness by phosphorylation^[74,77]^. Other indicators for the role of adrenergic hypophosphorylation in HFpEF come from the mild reduction in systolic function with prolonged contraction and relaxation indices, as well as the reduced responsiveness of the sinus node to adrenergic activation with either medications or exercise (chronotropic incompetence), which is improved by discontinuing beta-adrenergic antagonists^[23,78–81]^. Clinical studies tested the efficacy of phosphodiesterase type 3 (PDE3) inhibitors that slow the breakdown of cyclic AMP, with a subsequent increase in protein kinase A-dependent protein phosphorylation. These agents provided marked hemodynamic benefits in HFpEF, and improved the aforementioned clinical markers of hypophosphorylation, both at rest and during exercise^[82–85]^. It is, therefore, that PDE3 inhibitors can be viewed as adrenergic amplifiers that restore a more normal response that led to improvements in chronotropic response and 6-min walk distance, whereas amplification of the guanylyl cyclase pathway via PDE5 inhibition failed to exert a benefit^[84,86]^. The causes of the attenuated activation of the adrenergic pathway in humans are likely multifactorial and have not been specifically assessed and quantified. Phosphorylation also plays a key role in the regulation of cardiac contraction and relaxation, processes that are governed by myocyte calcium handling, which will be discussed next.

### Prolonged relaxation

The pathognomonic prolongation of the velocity and time indices of cardiac relaxation in HFpEF, such as - dP/dT and tau, is primarily caused by a slowed speed of diastolic calcium removal from the myofilaments^[87]^. Changes in the speed of relaxation are largely accounted for by an altered cardiac sarcoplasmic reticulum (SR) calcium pump (SERCA2a) activity. Accelerated calcium sequestration into the SR shortens relaxation (lusitropic effect) while increasing the SR calcium content, which, upon release from the SR, will result in a stronger contraction (inotropic effect)^[88]^. SERCA2a activity is primarily modulated by the protein kinase A-mediated phosphorylation of the SR protein phospholamban (PLB)^[89,90]^. Therefore, the slowed relaxation kinetics in HFpEF are accompanied by a slowing of the contraction velocity and a reduction in systolic function that is clinically detectable^[22,23,91]^. A hastened SR calcium uptake by PLB phosphorylation, i.e., by the aforementioned PDE3 inhibitors, will accelerate relaxation and contraction. Faster removal of calcium from the myofilament enhances ventricular suction and will lower filling pressures in all chambers.

Calcium is also crucial in mediating the effects of heart rate on the myocardium. Higher rates accelerate contraction and relaxation dynamics, independent of adrenergic activation. This innate myocardial property, referred to as the Treppe effect or force-frequency relationship, is the result of augmented cellular calcium cycling up to stimulation rates of 120–170 bpm, after which force development declines^[92,93]^. As the heart rate increases, so does relaxation velocity, which helps prevent incomplete relaxation. The myocardium of HFpEF patients abnormally retains cellular calcium, which adversely affects SR function, leading to prolonged relaxation and a build-up of diastolic tone^[22,23]^. While diastolic tone leads to smaller chamber volumes at rest, it is converted into higher filling pressures during exercise. Combined with chronotropic incompetence, these are key factors in the reduction of cardiac output reserve in HFpEF^[24–26,94,95]^. Aging generally leads to slower cellular calcium handling and relaxation through both SR-dependent and cell membrane mechanisms, as observed in humans and in experimental models. These changes are further compounded by the direct afterload-induced prolongation of relaxation in hypertension^[71,96]^. Varying cardiac cycle lengths, as the case in AF, will further perturb calcium handling. The inability to establish steady-state calcium handling may explain “cardiac irregulopathy”, and higher natriuretic peptide levels in AF^[97]^. As both contraction and relaxation are energy-consuming, it is plausible that the ability to exercise will be further limited by ischemia from coexistent coronary artery disease and microvascular rarefication^[69]^. Although prolonged relaxation and low heart rates are mechanistically linked, i.e., by hypophosphorylation, they have different pathomechanistic effects that independently contribute to diastolic dysfunction to warrant a separate discussion.

### Below optimal heart rates and conduction system disease

**Figure 3.**
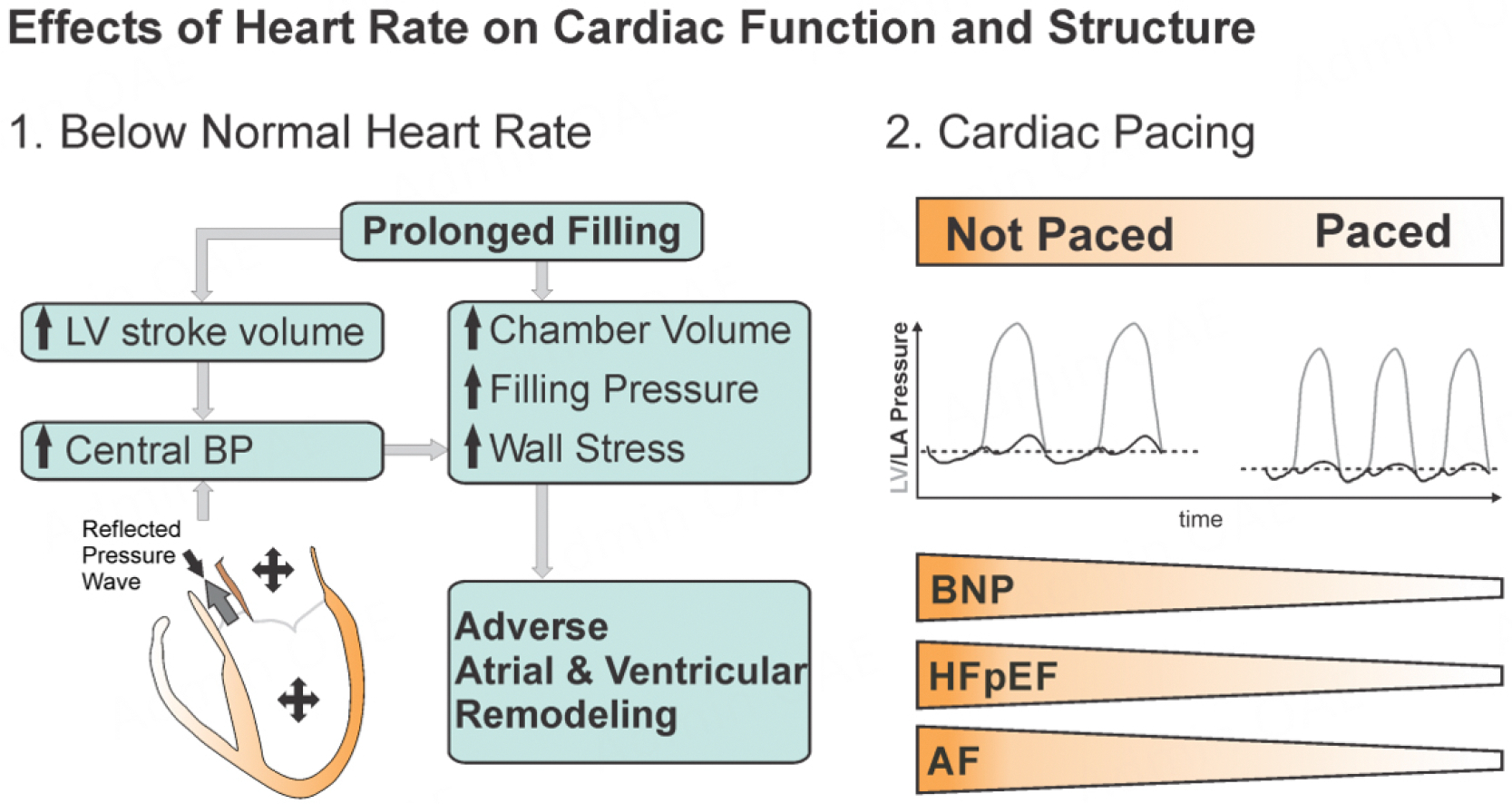
Effects of heart rate on cardiac function and structure.

As discussed earlier, latent and overt bradycardia leads to extended ventricular filling at higher pressures [Figure 3]^[39]^. It has been falsely promoted that prolonged filling by a slowing of heart rate would have beneficial effects^[98]^. This argument contributed to an overuse of beta-blockers, which remain the most commonly prescribed medication class in HFpEF and AF^[39,98,99]^. The natural deterioration in electrical conduction also contributes to diastolic dysfunction in HFpEF by reducing electromechanical synchrony and atrioventricular coupling^[36,100–102]^.

There are several reasons why below-normal heart rates, i.e., due to age-related sinus node dysfunction, may play a role in the development of HFpEF and AF. First, the regressive age-related ventricular remodeling is not matched by higher heart rates, to maintain optimal filling dynamics^[103]^. Second, large outcome studies and analyses confirmed that pharmacological heart rate lowering increases the risk for HFpEF and AF^[104–106]^. Third, the impaired chronotropic response of the sinus node to adrenergic activation during exercise decreases with age^[40,107]^. It is revealing that hospitalized patients with HFpEF tend to present with substantially lower heart rates compared to HFrEF, even when corrected for the use of beta-blockers, suggesting a general propensity toward low heart rates that directly promote HFpEF and AF^[95,108]^. This is also reflected in the large HFpEF clinical trials, where the baseline heart rates are typically between 69–71 *vs*. 80 bpm in the general population^[42,109]^. In this context, it is rarely considered that heart rates are inversely related to body size, which explains why heart rate is higher in childhood and is about 5 bpm higher in women compared to men^[110]^. By ignoring this allometric relationship and accepting heart rates of 60 bpm as normal, individuals with smaller body habitus, i.e., women, can be chronically exposed to heart rate-mediated filling pressure elevations^[42]^. We have argued elsewhere that heart rates around 70 bpm, as observed in HFpEF trials, are likely too low for a majority of age-remodeled hearts that, based on their altered physical properties, would function more optimally at higher heart rates^[111]^. In other words, many older adults have concealed bradycardia despite not meeting current criteria^[103]^. It is, therefore, prudent to generally avoid beta-blockers in the treatment of patients with preserved ejection fractions whenever possible, as they will worsen filling dynamics and promote dyspnea, the cardinal symptom of HFpEF^[39,99,112]^. Only heart failure patients with sinus rhythm and a reduced ejection fraction derive an unequivocal and substantial benefit from selective beta-receptor blockade^[113]^. In the following section, we will discuss the treatment targets that appear most promising in overt diastolic dysfunction manifested as AF and HFpEF.

## TREATMENT APPROACHES

AF treatment focuses on restoring sinus rhythm or controlling the ventricular rate. Because diastolic dysfunction is the prevailing cause for both HFpEF and AF, similar preventative strategies apply. However, most of the evidence basis for prevention has accrued in hypertension trials, such as the SPRINT trial^[47]^.

### Diuretic agents

Loop diuretics are a mainstay therapy to alleviate symptoms and prevent recurrent heart failure admissions. Although there is a lack of outcome studies, it is clear that dosing optimization in response to weight changes or signs and symptoms of heart failure improves outcomes. In a pivotal study of pulmonary artery pressure sensor-guided medication adjustments that predominantly centered around loop diuretics, 84 heart failure-related hospitalizations were reported in the pressure sensor intervention group (*n* = 270) compared with 120 in the control group (rate 0.32 *vs*. 0.44, hazard ratio [HR] 0.72, 95%CI: 0.60–0.85, *P* = 0.0002), independent of ejection fraction^[114]^. It appears likely that thiazides have additional benefits due to their antihypertensive properties. In the ALLHAT hypertension trial, chlorthalidone was superior to Lisinopril in preventing incident heart failure^[115]^.

### Antihypertensive agents

Given the prominent role of hypertension in the development of AF and HFpEF, blood pressure control is a central goal. Although there are no large comparative effectiveness trials, thiazides, angiotensin receptor blockers, angiotensin-converting enzyme inhibitors, or calcium channel blockers can be tailored to coexistent comorbidities. In our practice, we generally strive to normalize blood pressure. If this is not accomplished, we add mineralocorticoid receptor antagonists such as spironolactone to the regimen. The TOPCAT trial of spironolactone demonstrated a benefit in the participants from the Americas and finerenone confirmed this benefit^[116,117]^. If additional blood pressure control is required, we add sacubitril-valsartan to enhance blood pressure management and clinical efficacy, especially in patients with low normal ejection fractions^[49]^. If AF rate control is needed, i.e., heart rates that exceed 110 bpm, we avoid beta-blockers as they worsen diastolic function and preferentially use diltiazem or digoxin^[112,118]^. As discussed, in obesity-driven HFpEF and AF, it is already evident that GLP-1 agonists will be strikingly effective due to their significant ability to reduce blood pressure^[54]^.

### SGLT2 inhibitors

The first class of medications that demonstrated an unequivocal benefit in a majority of patients with HFpEF were the inhibitors of the renal sodium-glucose cotransporter 2 (SGLT2)^[50,52]^. These agents reduce the relative risk of HFpEF admissions by about 20% with minimal effect on mortality or surrogate markers of heart failure, such as quality of life. SGLT2 inhibitors have only minor blood pressure-reducing effects, and no single mechanism appears to fully explain their effectiveness.

### Outlook over the next decade

Even if beta-blocker de-prescription could be more widely implemented and pharmacological advances that include SGLT-2 inhibitors and GLP-1 agonists for weight loss are fully adopted, the residual risk for HFpEF and AF will remain high as the incurred decrement in diastolic function and changes in cardiac structure are not fully reversible. This is already evident in patients who were obese earlier in life^[119]^. The changing demographics toward an older population will require the additional development of targeted treatments that counteract the progression of diastolic dysfunction and the underlying structural abnormalities.

## PROSPECTIVE THERAPIES AND KNOWLEDGE GAPS

**Figure 4.**
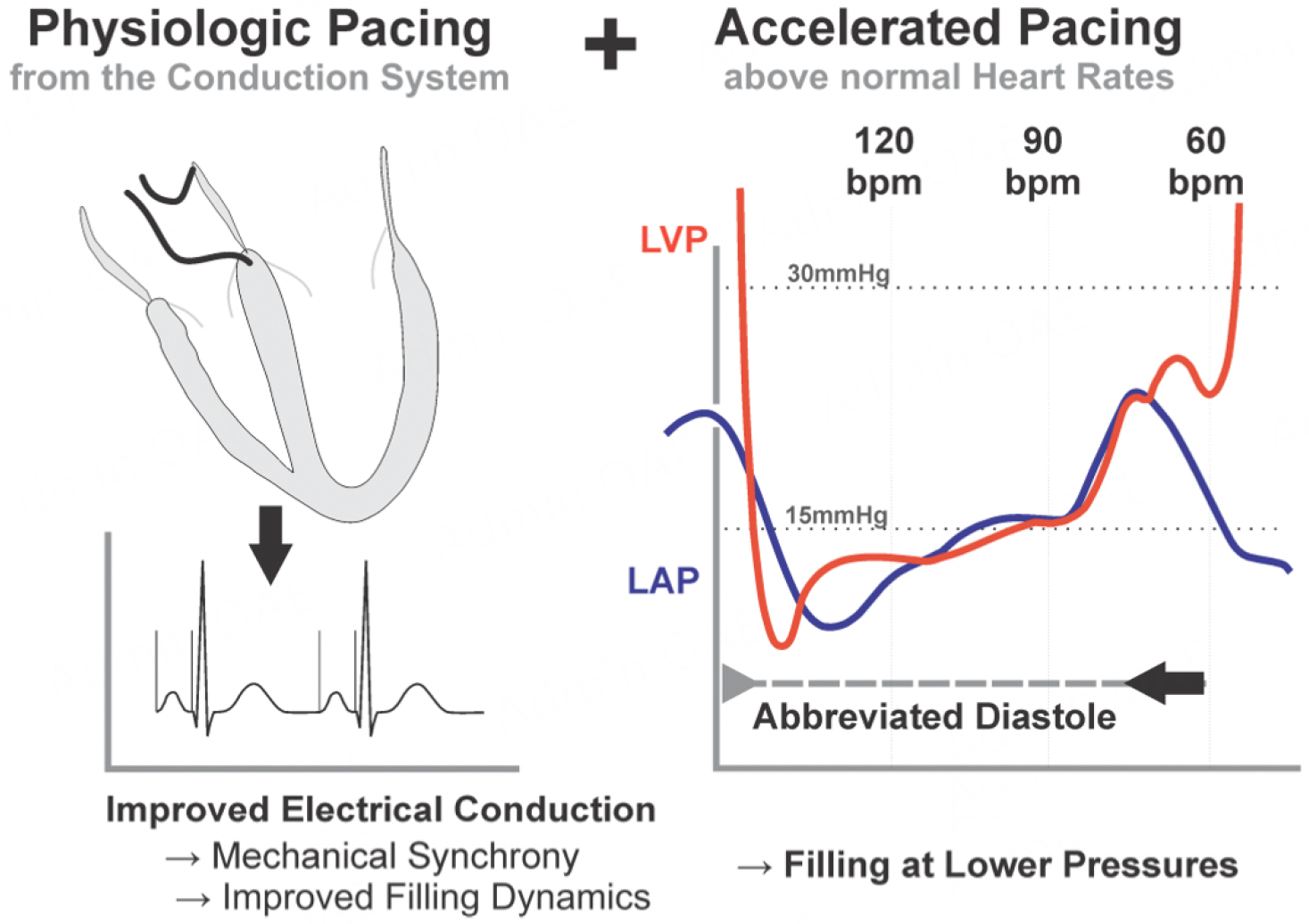
The combination of physiologic pacing, which recapitulates normal electrical conduction (short P wave and QRS duration) and enhances mechanical synchrony, and accelerated pacing, which abbreviates diastole and lowers filling pressures. The combination of physiologic and accelerated pacing has synergist effects.

A rational treatment of diastolic dysfunction is provided by the aforementioned PDE3 inhibitors^[120]^. These agents amplify the muted adrenergic signaling by increasing the half-life of cAMP, which in turn activates protein kinase A, thereby restoring cellular phosphorylation. This not only hastens relaxation by accelerated myofilament calcium removal, but also reduces the stiffness of titin. In addition, heart rate is increased, which is closely associated with lower filling pressures, symptom burden, and improved exercise capacity^[82,84,85]^. There may be, however, some hesitance to engage in long-term studies, as the results of historical HFrEF trials are inappropriately extrapolated to HFpEF. In the group of patients with the most advanced HFrEF, long-term use of PDE3 inhibitors improved symptoms but was associated with a modest increase in mortality^[121]^. However, safety studies of related PDE3 inhibitors did not find higher death rates in other patient groups^[122,123]^. Conceptually related is personalized accelerated physiologic pacing, which augments calcium handling by increasing heart rates above normal^[120,124]^. This approach has a similar efficacy with the added benefit that it helps correct coexisting conduction system disease without relying on adrenergic activation [Figure 4]^[120,125]^. Above-normal resting heart rates also induce a modest degree of stable eccentric remodeling with myocardial thinning^[126]^. This would improve not only ventricular capacitance but also compliance, which are prerequisites for a better exercise capacity^[30,127]^. This hemodynamic treatment approach also appears to unload the atria, which may reduce the propensity toward AF^[125,128–130]^.

It has been hypothesized and tested if an atrial shunt could provide a benefit by reducing left atrial pressures and symptoms. This approach, however, increases right-sided volume loads with unknown long-term effects and a risk of reproducing the untoward effects of naturally occurring atrial-level shunts. The clinical trials performed to date do not suggest a favorable effect in HFpEF and the benefits seen in subgroups may reverse with the aging-related progression of pulmonary resistance^[131,132]^. Similarly, splanchnic nerve denervation, which aims at reducing the excessive recruitment of dependent venous blood during exercise, has uncertain effects on cardiac performance and outcomes^[133]^.

At present, several trials are testing the effectiveness of anti-inflammatory agents under the premise that inflammation is a significant driver of HFpEF, i.e., by activating pro-fibrotic pathways. Although anti-inflammatory therapy targeting the interleukin-1β innate immunity pathway was demonstrated to significantly lower the rate of recurrent vascular events, it did not benefit heart failure^[134]^. As discussed, inflammatory pathway activation is also not evident in myocardial biopsies of patients with HFpEF^[66,67]^. Therefore, suppressing inflammation is unlikely to improve diastolic function.

Due to the high prevalence of the obesity-driven cardiometabolic HFpEF phenotype, it is predictable that GLP-1 agonists will markedly reduce the risk for HFpEF and AF. These benefits are in large part conveyed by the blood pressure reduction, but it is conceivable that the para-adrenergic effect of GLP-1 agonists may provide an additional more targeted benefit, i.e., by increasing heart rates^[135,136]^. Within the conceptual framework of accelerated diastolic aging, we predict that diastolic function and congestion will not revert to a healthy baseline and only transiently improve with GLP-1 agonists. In other words, patients will continue to be at a higher risk for HFpEF and AF compared to age-matched individuals who were never obese.

Undoubtedly, many more factors contribute to the deterioration in diastolic function. We have highlighted the ones we deem clinically most relevant and supported by the accruing evidence. Except for hypertension, which is the #1 accelerator of diastolic dysfunction, it is not possible to rank them. We remain optimistic that better and more relevant research can add more nuance to our understanding of the natural deterioration of human diastolic function, which in turn should improve the odds of finding effective treatments. Caution is advised with proclamations of a cure, which, in our opinion, is as realistic as immortality.

## SUMMARY

The age-dependent deterioration of diastolic function accelerated by obesity-driven hypertension is the main driver of the current HFpEF and AF epidemic. A better understanding of the shared pathophysiology will yield effective treatments that can ameliorate and possibly even slow the progression of diastolic dysfunction.

## Data Availability

Not applicable.
